# Comminuted Transarticular Fracture of the Middle Phalanx: A Non-standard Surgical Procedure

**DOI:** 10.7759/cureus.97754

**Published:** 2025-11-25

**Authors:** Oles Petrovych, Jakub Florek, Filip Georgiew, Patryk Kawa, Pawel Florek

**Affiliations:** 1 Department of Orthopedics and Traumatology, Rydygier Hospital, Brzesko, POL; 2 Faculty of Health Science, University of Applied Science, Tarnów, POL

**Keywords:** arthroplasty, endoprosthesis, fracture, pip, proximal interphalangeal joint

## Abstract

Fractures of the proximal interphalangeal (PIP) joint with fragment displacement should be promptly repaired after injury, though this does not ensure the return of pre-injury finger function. The most common surgical treatment involves open reduction and internal fixation (ORIF) or closed reduction and internal fixation (CRIF) using locking compression plate (LCP), intramedullary nails, or screws. In articular fractures of the hand and wrist, the surgical reduction of small fragments is generally less feasible to occur compared to long bone fractures. This article describes the case of a 58-year-old woman who presented to the emergency department with an injury to her right third finger. The injury resulted from a fall from a height. X-ray revealed a comminuted pilon fracture of the PIP joint. Due to the fracture type and age, the decision was made to utilize an arthroplasty technique using a semi-constrained 3S ORTHO prosthesis. The article describes the early results of treatment. A six-month follow-up examination revealed that the active flexion range of the PIP joint reached 75°, with no pain at rest or during function. The improvement in the patient's clinical condition allowed her to return to work.

## Introduction

Proximal phalanx fractures are common injuries of the hand, with estimated rates of 68 injuries per 100,000 persons per year. The highest incidence is reported in men between the ages of 30 and 57 years [1,2]. Hand fractures may account for 19% of all fractures diagnosed in emergency departments. Most of them involved the metacarpals, but as a group of bones, the combined phalanges were most commonly fractured. Approximately 14% involve the middle phalanges and often occur from an axial load and result in a fracture of the middle phalangeal at the dorsal or volar lip or both (pilon) [2,3]. The fifth finger is most frequently affected at 38% [3]. Of all finger joints, the proximal interphalangeal is the most vulnerable [4].

Fractures of the base of the phalange are classified based on the displacement of the fracture fragments. They most often displace by avulsion, i.e., through traction on tendons, ligaments, joint capsule, volar plate, or direct trauma [5,6]. Fractures are divided into dorsally displaced, palmar displaced, or pilon fractures. The most common surgical treatment involves open reduction and internal fixation (ORIF) or closed reduction and internal fixation (CRIF) using locking compression plate (LCP), intramedullary nails, or screws [2,7]. In the case of articular fractures of the hand and wrist, the possibility of surgical reduction of small fracture fragments is significantly lower compared to long bone fractures. Management strategies of proximal phalanx fractures attempt to balance anatomical, stable reduction with functional rehabilitation and the early range of motion [1]. According to Gianakos et al., regardless of the surgical technique used, three fundamental guidelines should be followed. These include the following: restore concentric gliding joint motion, restore joint surface congruity, and impart enough stability to the joint to allow the early range of motion [2].

Pilon-type injuries of the proximal interphalangeal (PIP) joint with numerous small fragments can pose a challenge for treatment with ORIF, CRIF, and dynamic distraction external fixation (DDEF). Treatment using these techniques can be unsatisfactory due to significant limitations of joint mobility and pain. Proximal interphalangeal joint arthroplasties provide viable alternatives to arthrodesis, osteotomy, and amputation in the appropriate patient [8]. Therefore, we attempted to use a 3S ORTHO semi-constrained endoprosthesis. This approach allows for rapid rehabilitation and, consequently, allows for a better range of motion. This prosthesis, thanks to its unique design, allows for the reconstruction of the anatomical axis of flexion in the PIP joint. The technology used allows for 100° of flexion. The primary stability associated with a titanium plasma coating guarantees durable osseointegration.

According to Richards et al., proximal interphalangeal (PIP) joint arthroplasty is a commonly performed procedure in patients with osteoarthritis [9]. Long-term results (over five years) are very good. According to Notermans et al., patient satisfaction was excellent in 19% of patients, good in 23%, reasonable in 24%, moderate in 14%, and poor in 20%. Seventy-three percent of patients would undergo the same procedure again [10].

The article presents a non-standard procedure in a patient with a comminuted, transarticular pilon fracture of the base of the middle phalanx of the PIP joint treated by a 3S ORTHO semi-constrained endoprosthesis.

## Case presentation

**Figure 1:**
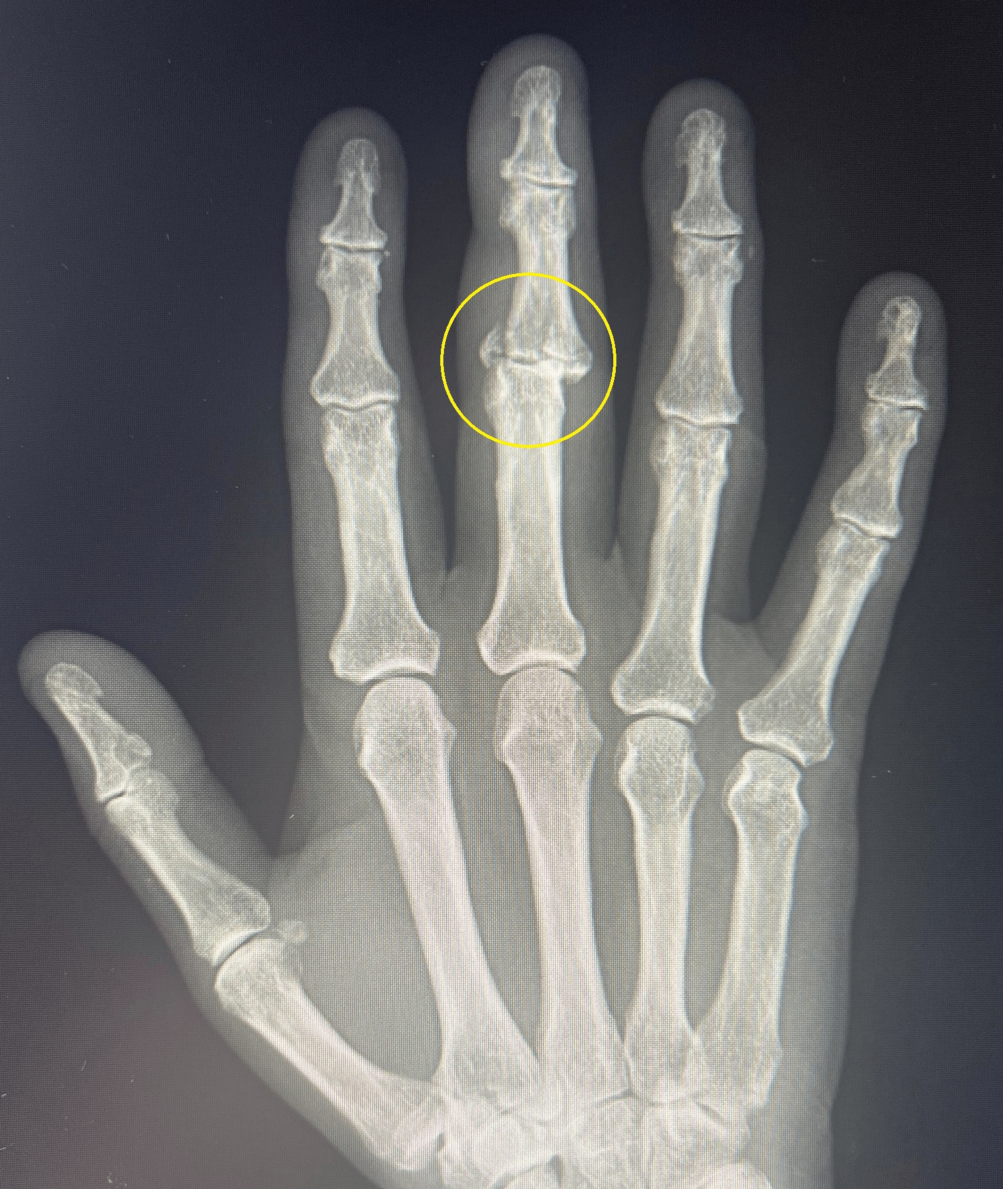
Comminuted pilon fracture of the PIP joint (X-ray in the AP projection) PIP: proximal interphalangeal

**Figure 2:**
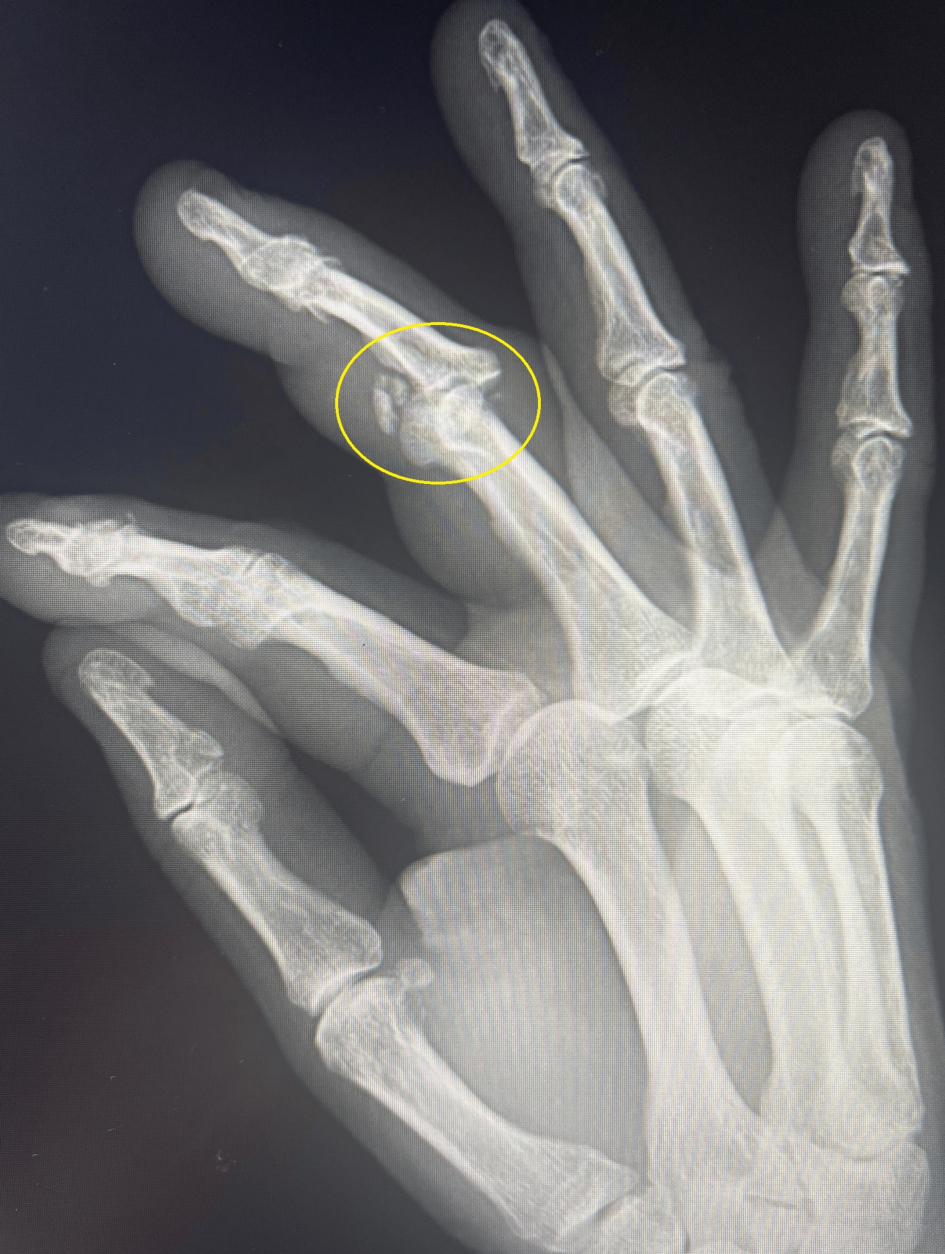
Comminuted pilon fracture of the PIP joint (X-ray in Robert's projection) PIP: proximal interphalangeal

This article describes the case of a 58-year-old woman who presented to the emergency department with an injury to her right third finger. The injury resulted from a fall from a height. X-ray revealed a comminuted pilon fracture of the PIP joint (Figures 1, 2).

**Figure 3:**
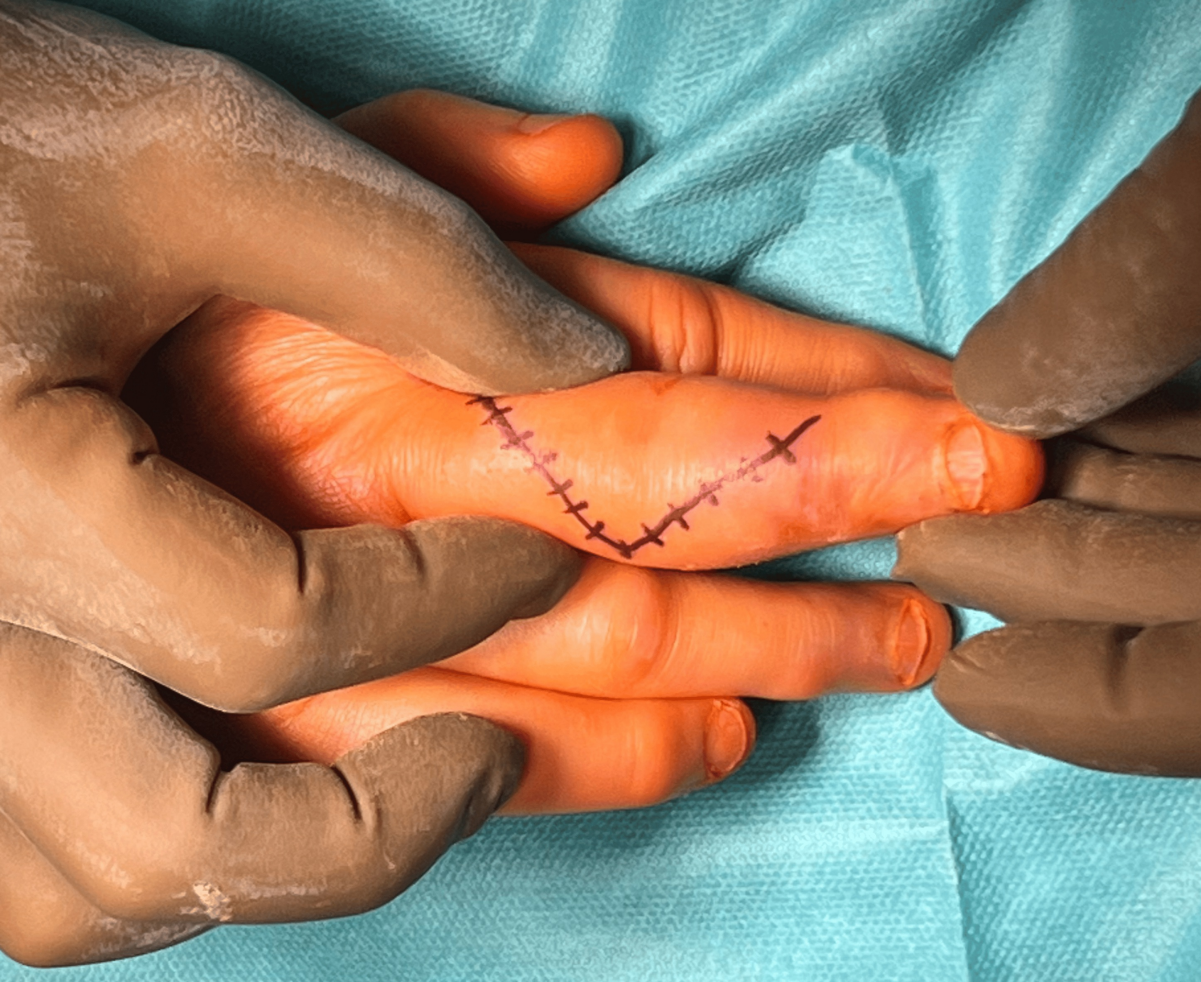
Course of surgical incision

The patient was initially treated in the hospital emergency department. A follow-up visit to the orthopedic clinic was recommended, which qualified the patient for surgical treatment. Due to the fracture type and age, the decision was made to utilize an arthroplasty technique using a semi-constrained prosthesis. The surgical procedure was performed via a dorsal approach using a dorsal oblique incision at the level of the proximal and middle phalanges of the third finger of the hand (Figure 3).

**Figure 4:**
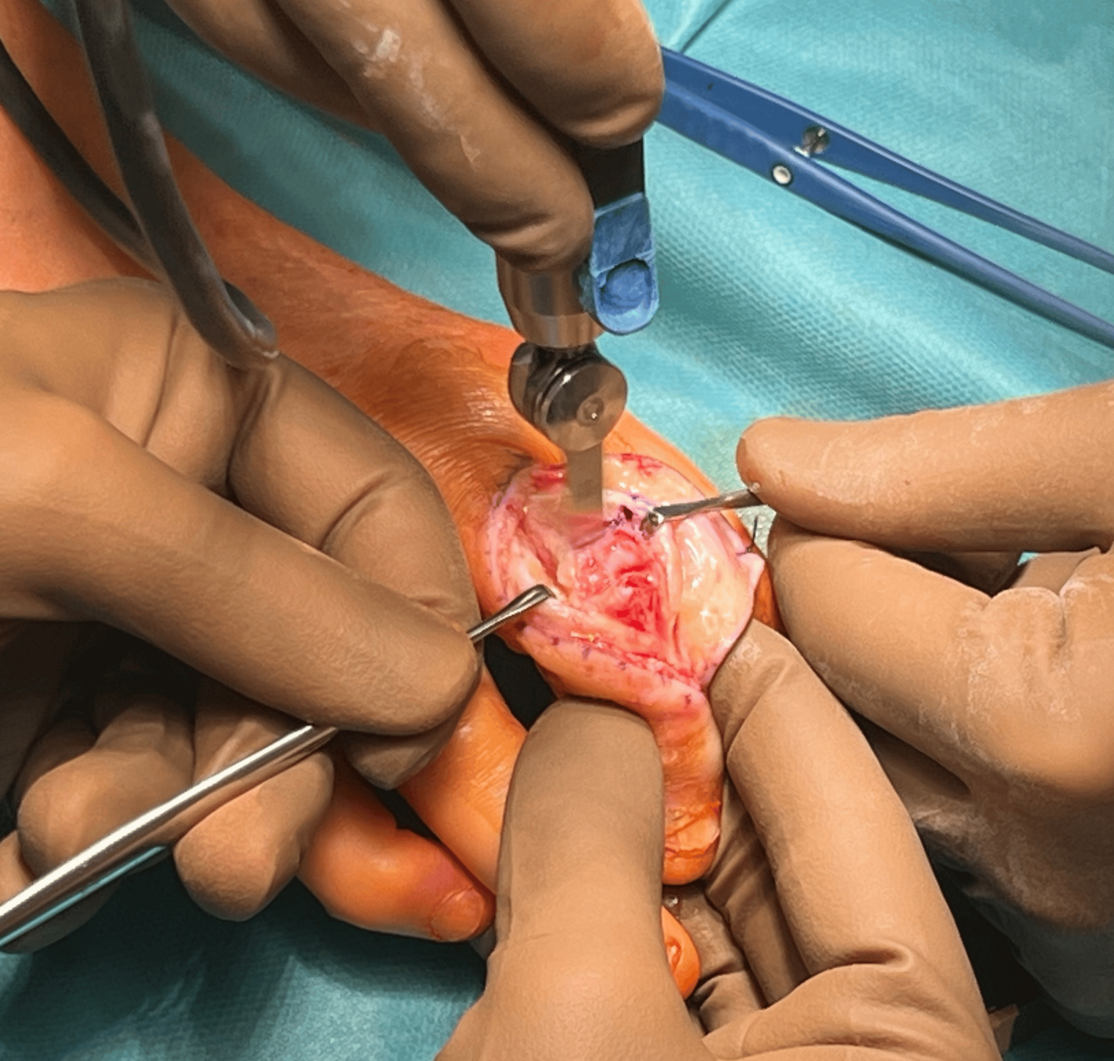
Preparation of joint surfaces for the implantation of a prosthesis into the PIP joint PIP: proximal interphalangeal

After thorough tissue dissection, the central band was reached and cut longitudinally to reach the PIP joint capsule. The PIP joint capsule was also cut longitudinally, revealing a comminuted, transarticular, fragmented fracture of the proximal end of the middle phalanx of finger III (Figure 4).

**Figure 5:**
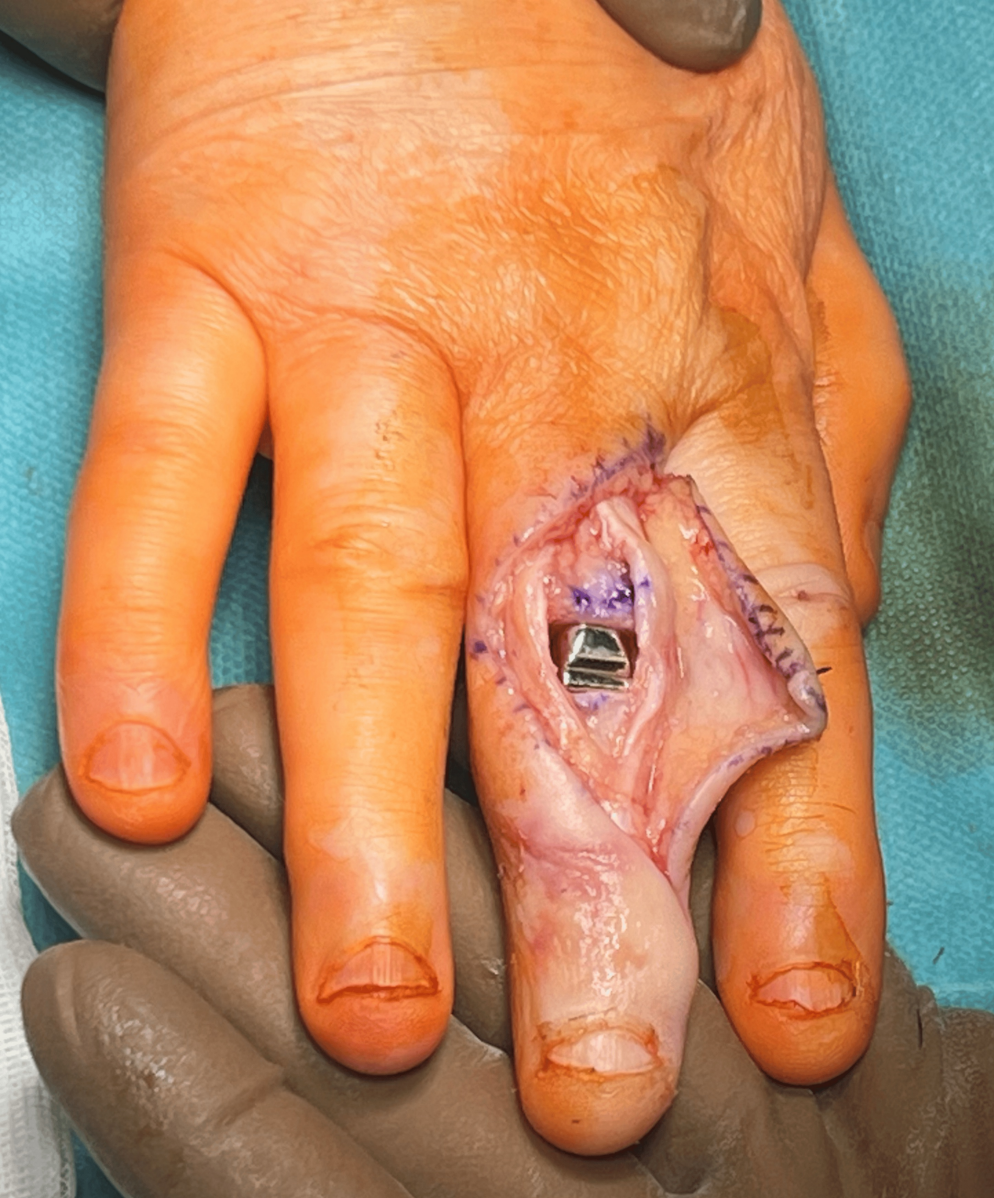
Properly implanted prosthesis for the PIP joint of the third finger of the hand PIP: proximal interphalangeal

Initially, a reduction attempt was made, but due to the extensive fragmentation and advanced degenerative changes in the articular surface of the distal phalanx, the decision was made to implant the 3S ORTHO prosthesis. Using a special sizer, the articular surfaces of the proximal and middle phalanges were prepared. Screws securing the prosthesis to the proximal and middle phalanges were planned and implanted, and then, a 3S ORTHO semi-constrained prosthesis connector was inserted, achieving a stable PIP joint (Figure 5).

**Figure 6:**
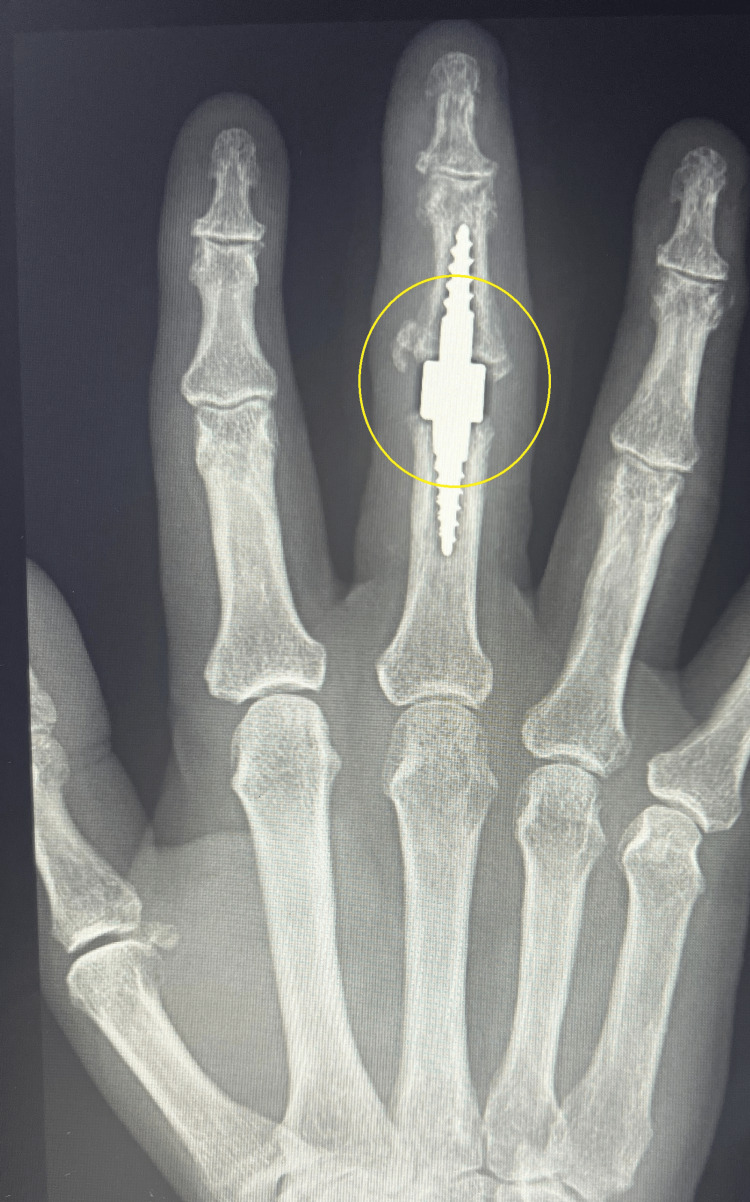
Control X-ray in AP projection obtained after surgery

**Figure 7:**
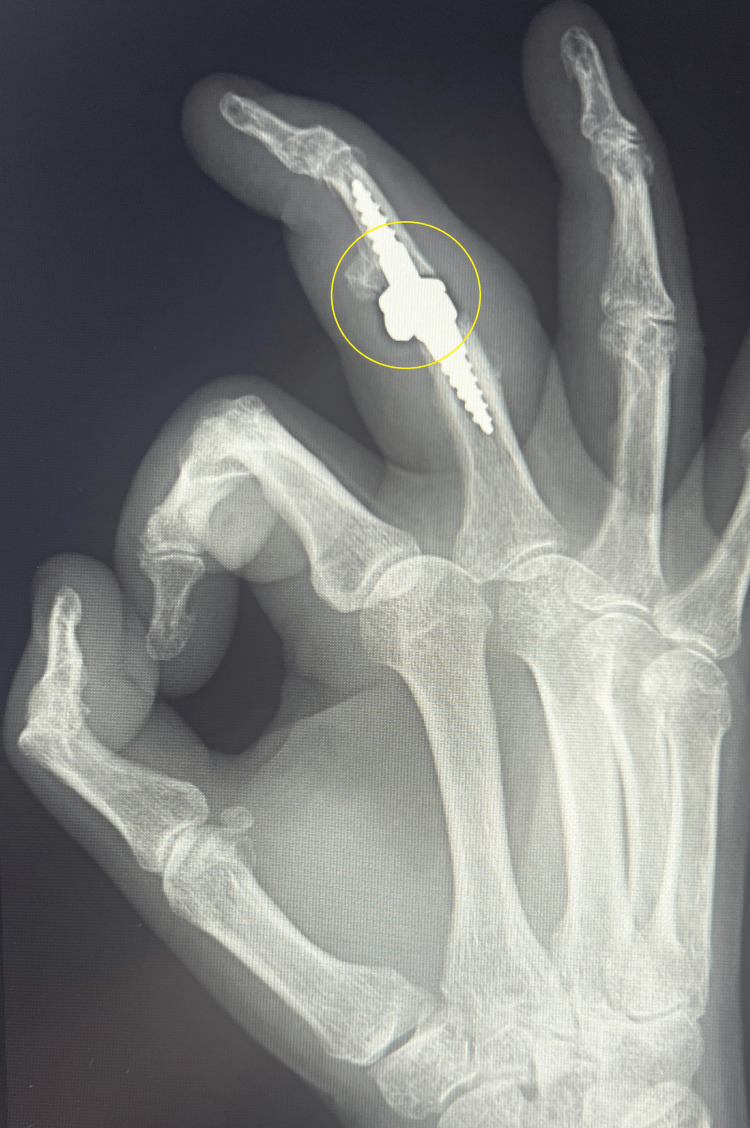
Control X-ray in Robert's projection obtained after surgery

Intraoperative X-ray confirmed the proper preparation of the joint surfaces and the correct positioning of the prosthetic implants. The joint capsule was sutured, and a reconstruction of the central band of the middle finger was performed. Layered sutures and a sterile dressing were applied. A palmar plaster splint was implanted for 10 days, encompassing the fourth and third fingers. After the procedure, a control X-ray was performed, which showed the correct positioning of all prosthesis elements (Figures 6, 7).

On the 12th postoperative day, the sutures were removed, and rehabilitation began. Until the postoperative wound healed, the limb was fitted with a palmar plaster splint, encompassing fingers III and IV. The rehabilitation program involved the patient performing exercises at home (following instructions from a physiotherapist). Initially, it included passive and active exercises for both the operated finger and the other fingers, with particular emphasis on flexor tendon gliding exercises. Over time, resistance exercises and activities of daily living were introduced.

**Figure 8:**
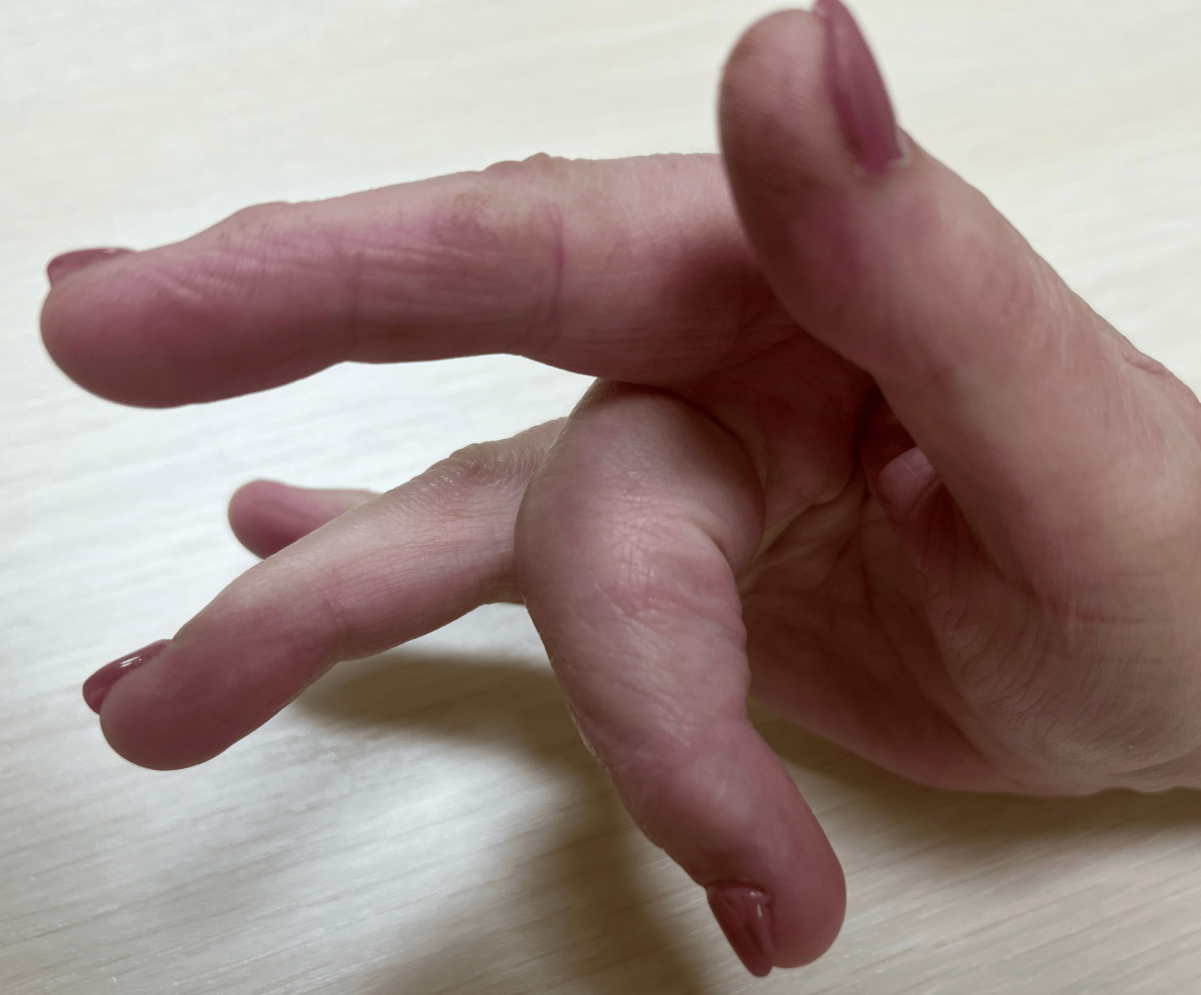
Flexion range of motion in the PIP joint of the third finger six months after surgery PIP: proximal interphalangeal

**Figure 9:**
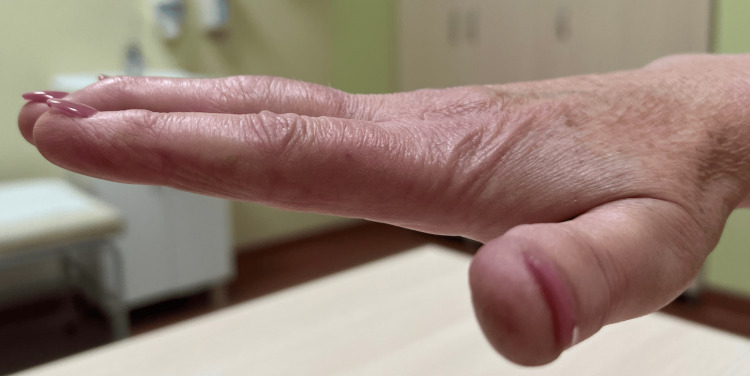
Extension range of motion in the PIP joint of the third finger six months after surgery PIP: proximal interphalangeal

After six months, the clinical condition of the operated hand was assessed, paying particular attention to the range of motion of the operated finger. The clinical examination revealed that the active flexion range of the PIP joint reached 75°, with no pain at rest or during physical activity (Figures 8, 9). The improvement in the patient's clinical condition allowed her to return to work. Six months after the procedure, she was fit to work. It is worth noting that the patient works as a dental prosthetist, a profession that requires high dexterity and precision of hand movements.

**Figure 10:**
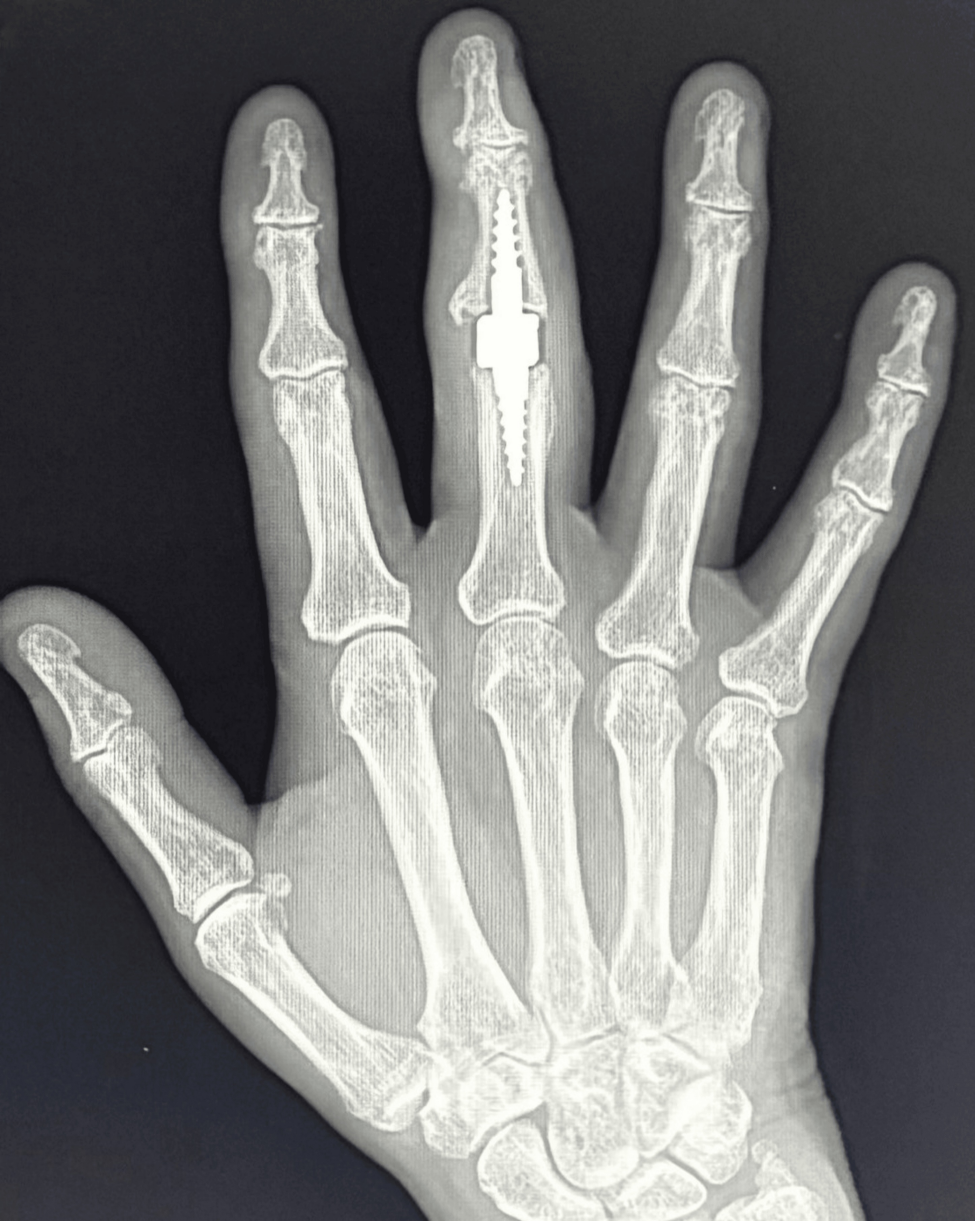
Control X-ray in AP projection six month after surgery

**Figure 11:**
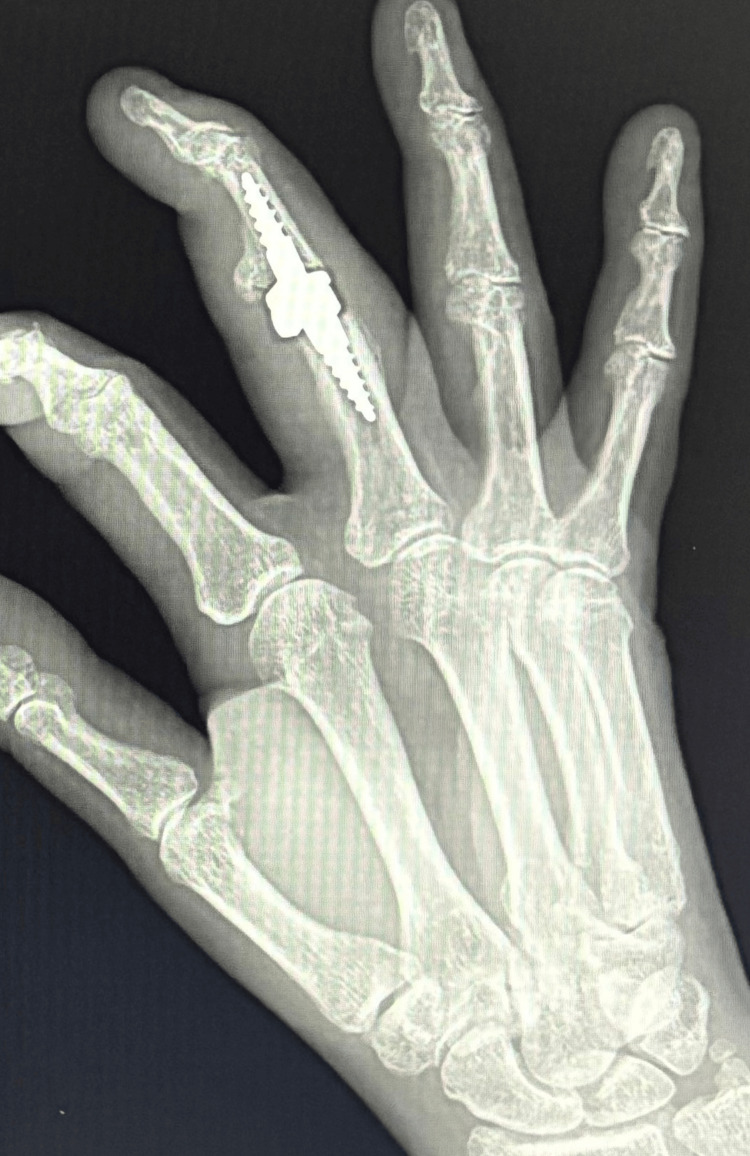
Control X-ray in Robert's projection six month after surgery

A control X-ray taken at six months showed the correct positioning of the prosthesis components, progressive osteointegration, no signs of loosening, no periarticular ossifications, and the correct axis of the third radius of the right hand (Figures 10, 11).

## Discussion

Transarticular fractures can pose a significant therapeutic challenge due to the high risk of complications and limited treatment effectiveness. This is due to the fact that they can be accompanied by significant soft tissue damage, which impedes postoperative wound healing, leads to impaired vascularization and nutrition of the affected limb, disrupts bone union, and, in the most severe cases, leads to amputation [4]. The choice of treatment method depends on several factors. In patients with non-displaced or slightly displaced fractures, conservative treatment can be used. A drawback of this treatment method is that the PIP joint quickly contracts due to immobilization [11,12]. For unstable fractures, the treatment of choice is surgery, such as ORIF, CRIF, and dynamic distraction external fixation (DDEF). In cases of significant joint damage, joint arthrodesis may be considered [7,11,12]. Pilon fractures have the worst treatment outcomes due to severe cartilage damage. Patients often continue to report pain and a lack of satisfactory joint mobility after surgery. The PIP joint is highly susceptible to stiffening, even after short-term immobilization. This fact is also pointed out by Raducha and Weiss, who state that PIP joint arthroplasties provide excellent pain relief and patient satisfaction, but patients should be cautioned not to expect an improvement in motion postoperatively [8]. Gianakos et al. suggest that patients undergoing treatment for PIP fractures and fracture-dislocations should be made aware that they are not likely to regain a full range of motion [2].

Darwish et al. reviewed the literature published between 1990 and 2021 on PIP joint arthroplasty. Twelve studies covering a total of 412 PIP joints were included in the analysis. The achieved range of motion depended on the type of prosthesis material and was as follows: metal implants, 66.6°; silicone, 55.8°; and pyrocarbon, 46.4°. Pain intensity assessed on a visual analog scale (VAS) also depended on the type of prosthesis. Silicone implants achieved the best score at 1.2, pyrocarbon at 2.6, and metal at 3.9. Complication rates were also lowest for silicone implants at 11.3%, compared to 18.5% for pyrocarbon prostheses and 22.4% for metal prostheses. Prosthesis survival did not differ significantly between groups [13]. Forster et al. performed a systematic review and meta-analysis of the incidence of complications, reoperations, and revisions after proximal interphalangeal joint replacement using pyrocarbon, metal-polyethylene, and silicone implants. Implant-related complications were associated with 14%, 10%, and 11% of pyrocarbon, metal-polyethylene, and silicone implants, respectively, but these rates did not differ significantly. The lowest reoperation rate was observed for silicone implants at 1%. The rate was 7% for pyrocarbon implants and 10% for metal-polyethylene implants. Revision rates were similar for all implant types [14]. Richards et al.'s study demonstrates the durability of PIP joint replacement of the index finger, providing pain relief and patient satisfaction. In their study group, 87% of the patients opted for reoperation, and 97% preferred this option over arthrodesis. The authors strongly advocate silicone arthroplasty for painful osteoarthritis of the PIP joint of the index finger. At the same time, they strongly question the previous tendency to recommend fusion as the default procedure in such cases [9]. A valuable observation has been described in the article by Forster et al., who argue that resurfacing implants may be more effective in correcting unstable or deviated PIP joints, although they are associated with a higher risk of reoperation [14].

According to Notermans et al., patient-reported outcomes improve primarily within the first year and remain stable for five years or longer [10]. Therefore, in our case report, we described only early treatment outcomes. Although the patient's full range of motion was not restored, the undoubted success of this method is the fact that she returned to her previous professional activities, which she was able to perform without concomitant pain.

## Conclusions

The implantation of a 3S ORTHO semi-constrained endoprosthesis in a patient with a comminuted, transarticular fracture of the middle phalanx enabled a very good range of flexion motion in the PIP joint. A six-month follow-up examination revealed that the active flexion range of the PIP joint reached 75°, with no pain at rest or during function. The improvement in the patient's clinical condition allowed her to return to work. These results reflect the early follow-up period. The conclusions presented in the article are based on a single clinical case. Therefore, they have limitations and the need for further study to validate the effectiveness of this non-standard procedure.
